# Creatinine-Derived Composite Indices for Improved Mortality Prediction in Dogs with Acute Pancreatitis

**DOI:** 10.3390/ani16152283

**Published:** 2026-07-23

**Authors:** Alba Díaz González, Lorena Espadas-González, José Ignacio Cristóbal Verdejo, Carlos Martínez Zafra, Eva María Pérez-Merino

**Affiliations:** Departamento de Medicina Animal, Hospital Clínico Veterinario, Facultad de Veterinaria, Universidad de Extremadura, Avenida de la Universidad s/n, 10003 Cáceres, Spain; albadiazglez@gmail.com (A.D.G.); lorenaeg@unex.es (L.E.-G.); jicristob@unex.es (J.I.C.V.); carlosmarzafra1d@gmail.com (C.M.Z.)

**Keywords:** canine acute pancreatitis, biomarkers, mortality, neutrophil-to-lymphocyte ratio, neutrophil-to-creatinine index, creatinine-to-albumin ratio

## Abstract

In dogs, acute pancreatitis (AP) is frequently associated with systemic inflammation and renal dysfunction, both of which may adversely affect outcomes. This retrospective cohort study evaluated the prognostic utility of the creatinine-to-albumin ratio (CAR), neutrophil–creatinine index (NCI), and neutrophil-to-lymphocyte ratio (NLR) for predicting in-hospital mortality. One hundred and thirty-four dogs diagnosed with AP and forty healthy controls were included. All indices were calculated from routine hematological and biochemical data obtained at admission before treatment initiation. Prognostic performance was assessed using logistic regression, receiver operating characteristic (ROC) curve analysis, and comparison of ROC curves using the DeLong test. The results showed that all indices were significantly higher in dogs with AP than in healthy controls and were further increased in non-survivors. The mortality prevalence increased significantly across CAR and NCI percentiles, whereas no consistent association was observed for the NLR. CAR (AUC = 0.747, cutoff > 0.48, OR = 5.63) and NCI (AUC = 0.747, cutoff > 26, OR = 7.08) showed the highest discriminatory ability, followed by serum creatinine (AUC = 0.734, cutoff > 1.19 mg/dL, OR = 6.08). A ROC curve comparison revealed no significant differences between creatinine and CAR (*p* = 0.479), creatinine and NCI (*p* = 0.712), or CAR and NCI (*p* = 0.982). However, the NCI demonstrated significantly greater discriminatory performance than the NLR (AUC = 0.619; *p* = 0.021). In multivariable analyses, CAR and NCI remained independently associated with the outcome after adjustment for the NLR. These findings suggest that CAR and NCI may provide useful prognostic information in dogs with acute pancreatitis. Because they are derived from routinely available laboratory parameters, they may represent practical tools for early risk stratification at hospital admission.

## Simple Summary

Acute pancreatitis is common in dogs and has the potential to cause severe, life-threatening complications. Some dogs recover quickly, while others develop severe complications and may not survive. Veterinarians need simple and reliable tools available at the time of hospital admission to identify dogs at a higher risk of death and adjust treatment accordingly. This study evaluated whether combining routine blood test results could improve the early prediction of outcome in dogs with acute pancreatitis. We studied 134 client-owned dogs diagnosed with acute pancreatitis and compared them with healthy dogs. Two combined measurements based on kidney function and systemic inflammation, the creatinine-to-albumin ratio and the neutrophil-to-creatinine index, were calculated when the dogs arrived at the hospital. Dogs with higher values of these indices were more likely to die during hospitalization. These combined measurements predicted the risk of death better than commonly used individual blood values or inflammatory markers. Because these indices use simple laboratory parameters that are already measured in daily practice, they can be easily applied without additional cost or specialized equipment. Their use may help veterinarians identify high-risk patients earlier, improve clinical decision-making, and ultimately contribute to better management and welfare of dogs with acute pancreatitis.

## 1. Introduction

Canine acute pancreatitis (AP) is characterized by the abrupt onset of pancreatic inflammation, histologically marked by neutrophilic infiltration and, in severe instances, pancreatic necrosis [1]. AP is a prevalent condition in dogs, with reported mortality rates ranging from 27% to 42% [2]. Clinically, the disease presents with non-specific signs, most commonly including anorexia, vomiting, diarrhea, and abdominal pain [3]. Although many affected dogs experience mild disease and recover within a few days, a subset progress to systemic inflammatory response syndrome (SIRS), which may result in multiple organ failure and death [1]. Consequently, early prognostic assessment is essential to guide therapeutic intensity and clinical monitoring.

Acute kidney injury (AKI) is the most frequently reported complication of AP, with up to 26% of patients developing azotemia [3]. Inflammatory cell infiltration and the release of pro-inflammatory mediators play a major role in AKI pathogenesis and the subsequent increase in serum creatinine concentration [4,5,6]. Creatinine is a readily available and cost-effective biomarker and has consistently been associated with increased mortality in canine AP [3,7,8,9].

In human medicine, composite indices combining creatinine with other routinely measured parameters have been developed to improve prognostic accuracy at admission. One such index is the creatinine-to-albumin ratio (CAR). Hypoalbuminemia has been associated with increased short-term mortality in canine [10] and human AP [11,12], reflecting the acute phase response and altered hepatic protein synthesis secondary to systemic inflammation [13,14]. While CAR has proven prognostic value in human AP [11,12], its clinical utility has not yet been evaluated in dogs.

Another recently proposed index with demonstrated value in human AP prognosis but that has not been studied in dogs is the neutrophil–creatinine index (NCI), which integrates inflammatory activation and renal dysfunction [15]. Neutrophils are central mediators in the pathogenesis of AP and frequently increase in early disease stages [16]. The neutrophil count forms the basis of the neutrophil-to-lymphocyte ratio (NLR), one of the most extensively studied inflammatory markers, although the published results regarding its prognostic utility in canine AP are inconsistent [7,17].

Therefore, this study was conducted to evaluate the effectiveness of CAR and NCI as predictors of mortality in dogs with acute pancreatitis and to compare their prognostic accuracy with that of their components and the NLR. We hypothesized that these creatinine-based composite indices provide superior discriminatory ability for predicting in-hospital mortality, offering a valuable tool for detecting early risk of death in clinical practice.

## 2. Materials and Methods

This retrospective observational cohort study was conducted at the Veterinary Teaching Hospital of the University of Extremadura (VTH UEx). Medical records from January 2021 to January 2025 were reviewed, and dogs diagnosed with acute pancreatitis (AP) were identified. Ethical review was waived due to the retrospective nature of the study. All data were collected from patients whose owners had provided prior written informed consent for research purposes at hospital admission.

The diagnosis of AP at admission required the acute onset of the following clinical signs: vomiting, diarrhea, nausea, anorexia, abdominal pain, or lethargy; the presence of two or more ultrasonographic findings consistent with AP: pancreatic enlargement, hypoechoic pancreatic parenchyma, hyperechoic surrounding mesenteric fat, or peripancreatic fluid accumulation; and a canine pancreatic lipase (cPL) concentration above the reference range (cPL > 160 U/L) [14,18,19].

The inclusion criteria were the availability of complete hematological and serum biochemical data obtained within 24 h of presentation and prior to the administration of any treatment at the hospital (including fluid therapy), together with a minimum follow-up period of one month. Due to the retrospective design and the clinical presentation of the patients, a standardized fasting status could not be guaranteed. Dogs were excluded if their medical records were incomplete or if they had concurrent diseases, previous exposure to endogenous or exogenous glucocorticoids, recurrent episodes of AP, or persistence of clinical signs for more than one week. To minimize potential confounding related to renal biomarkers, dogs with a known history of chronic kidney disease were also excluded.

Because reference intervals for the evaluated ratios have not been established in veterinary medicine, a control group of forty clinically healthy dogs was included to determine baseline values and confirm ratio alterations in dogs with AP. These dogs (20 males and 20 females) were admitted for routine sterilization procedures and were considered healthy based on clinical history, physical examination, and hematological and serum biochemical analyses.

Collected data included signalment (age, sex, and breed), length of hospitalization (days), and outcome. Disease severity was assessed at admission using the canine acute pancreatitis clinical severity index (CAPCSI), which evaluates cardiovascular, respiratory, gastrointestinal, and blood pressure parameters. CAPCSI scores were calculated according to previously published criteria and recorded as a continuous variable [20].

Upon presentation, all dogs underwent a complete physical examination, complete blood count analysis (ProCyte DX^®^, IDEXX Laboratories, Westbrook, ME, USA), serum biochemical analysis (Spin200E, Spinreact^®^, Barcelona, Spain), and cPL measurement (FUJI DRI CHEM 4000i, Fujifilm^®^, FUJIFILM Corporation, Tokyo, Japan). According to the manufacturer’s reference intervals, cPL concentrations < 160 U/L were considered indicative of a low likelihood of pancreatitis, whereas values > 160 U/L were consistent with a high likelihood of AP [14,18,19].

Laboratory variables recorded for analysis included absolute neutrophil and lymphocyte counts (×10^3^/µL), serum albumin concentration (g/dL), and serum creatinine concentration (mg/dL). All laboratory data are reported in these conventional units to facilitate clinical interpretation and comparison with the existing veterinary literature and established severity scores.

The evaluated hematological and biochemical-derived indices were calculated as follows:

- NLR: absolute neutrophil count divided by absolute lymphocyte count;
- CAR: serum creatinine concentration divided by serum albumin concentration [11];
- NCI: absolute neutrophil count multiplied by serum creatinine concentration [15].

The outcome measure was survival to hospital discharge. Dogs were classified as survivors if they were discharged alive from the hospital and as non-survivors if they died or were euthanized during hospitalization. In accordance with the ethical standards of our institution, euthanasia was never chosen for financial reasons. It was performed strictly based on clinical criteria, to prevent further refractory suffering despite intensive medical therapy.

Statistical analyses were performed using IBM SPSS Statistics for Windows, version 29.0 (IBM Corp., New York, NY, USA). The normality of continuous variables was assessed using the Kolmogorov–Smirnov test. As data were not normally distributed, the results are reported as median and range (minimum–maximum).

Comparisons of quantitative variables between dogs with AP and healthy controls, as well as between surviving and non-surviving dogs with AP, were performed using the Mann–Whitney U test. Categorical variables, including clinical and ultrasonographic findings, were compared between survivors and non-survivors using the chi-squared test.

Dogs with AP were categorized into percentile groups for CAR, NCI, and NLR. Mortality prevalence within each percentile was expressed as a percentage. The association between increasing marker percentiles and mortality was evaluated using the chi-squared test, and the strength and direction of this association were further assessed using Somers’ D statistic.

Receiver operating characteristic (ROC) curve analysis was used to assess the ability of individual analytes and derived indices to discriminate between survivors and non-survivors. The area under the ROC curve (AUC) was calculated, and optimal cutoff values were determined using the Youden index. The prognostic effect of values above the identified cutoffs was expressed as odds ratios (ORs) with 95% confidence intervals (CIs). The differences between the areas under the ROC curves (AUCs) were evaluated using the DeLong test.

Univariate logistic regression analysis was used to identify variables significantly associated with survival. Multivariable logistic regression models were subsequently constructed to assess the independent association between selected biomarkers and outcome, with variable inclusion guided by biological plausibility and clinical relevance.

Correlations between hematological-derived indices and clinical severity (CAPCSI) were evaluated using Spearman’s rank correlation analysis for non-parametric variables.

## 3. Results

### 3.1. Study Population

**Figure 1:**
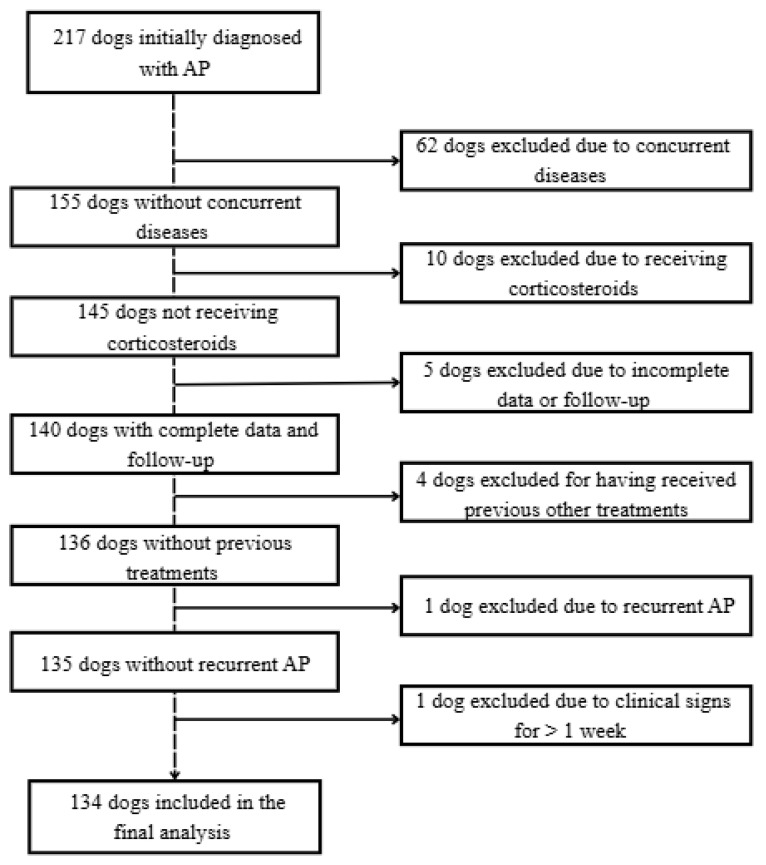
Flowchart of the case selection and exclusion process for dogs diagnosed with acute pancreatitis (AP). Dashed arrows indicate the progression of dogs through the inclusion process, whereas solid arrows indicate exclusions and the reasons for exclusion at each stage.

The hospital management software initially identified 217 dogs diagnosed with AP. Of these, 62 dogs had concurrent diseases documented in their medical records, 4 had received previous treatments, 10 were receiving corticosteroids, 5 lacked complete data or follow-up, 1 had recurrent AP, and 1 dog presented clinical signs for more than one week (Figure 1). All these cases were excluded, resulting in a final study population of 134 dogs.

Twenty-nine dogs were mixed-breed, and 105 were purebred. Yorkshire Terrier (n = 25) and Dachshund (n = 15) were the most frequently represented breeds. Seventy-five dogs were male, and fifty-nine were female, with a median age of 10 years (range 1–18).

The healthy control group comprised 40 dogs (20 males and 20 females) with a median age of 4.2 years (range 2–10). Seventeen were mixed-breed, and twenty-three were purebred, with Border Collie (n = 11), Dachshund (n = 7), and Yorkshire Terrier (n = 6) being the most represented breeds. There were no significant differences between dogs with AP and healthy dogs regarding sex distribution (*p* = 0.24) or breed (*p* = 0.12); however, healthy dogs were significantly younger than dogs with AP (*p <* 0.0001).

### 3.2. Clinical and Ultrasonographic Findings

Vomiting was observed in 130 of 134 dogs with AP (97%), diarrhea in 85 (63.4%), anorexia in 122 (91%), abdominal pain in 120 (89.5%), lethargy in 36 (26.8%), and signs compatible with nausea in 35 (26.1%).

Ultrasonographic examination revealed pancreatic enlargement in 62 dogs (46.3%). A hypoechoic pancreatic parenchyma was detected in 109 dogs (81.3%), whereas a hyperechoic pancreatic parenchyma was observed in 17 dogs (12.6%). Heterogeneous pancreatic echotexture was present in 62 dogs (46.3%), and peripancreatic free fluid was detected in 6 of 134 dogs (4.5%). Pancreatic cysts or pseudocysts were identified in four dogs (2.9%). None of the dogs exhibited hypoechoic mesentery surrounding the pancreas; however, 115 dogs (85.8%) presented hyperechoic mesentery.

The median interval between the onset of clinical signs and admission was 3 days (range 0.5–7). Median serum cPL concentration at admission was 1181 U/L (range 172–2358).

### 3.3. Comparison Between Dogs with AP and Healthy Dogs

Comparative results between dogs with AP and healthy controls are shown in Table 1. Dogs with AP had significantly higher neutrophil counts, serum creatinine concentrations, CAR, NCI, and NLR values, as well as significantly lower lymphocyte counts and serum albumin concentrations than healthy dogs (all *p <* 0.0001).

### 3.4. Renal Status at Admission

Based on the serum creatinine cut-off values established by the IRIS guidelines for AKI, 57 dogs (42.5%) were classified as azotemic (creatinine > 1.6 mg/dL), and 77 dogs (57.5%) as non-azotemic (creatinine ≤ 1.6 mg/dL). Azotemic dogs were significantly older than non-azotemic dogs (*p* < 0.0001) and showed significantly higher creatinine concentrations, CAR, NCI, and mortality rates (all *p <* 0.0001). No significant differences were observed between azotemic and non-azotemic dogs regarding neutrophil count, albumin concentration, or NLR (Table S1).

### 3.5. Outcome Analysis

Dogs with AP were hospitalized for a median of 3 days (range 1–4) and had a median CAPCSI score of 4 (range 2–8). Eighty-three dogs (61.9%) survived to discharge, whereas 51 dogs (38.1%) died or were euthanized during hospitalization.

No significant differences between survivors and non-survivors were observed for age (11.3 [3–17] vs. 10.7 [1–18] years; *p* = 0.18), breed (*p* = 0.34), sex distribution (*p* = 0.38), duration of clinical signs before admission (3 [0.5–7] vs. 4 [1–7] days; *p* = 0.35), serum cPL concentration (1153 [172–1940] vs. 1224 [386–2358] U/L; *p* = 0.52), fre-quency of clinical signs (*p* = 0.10), or ultrasonographic findings (*p* = 0.26).

CAPCSI scores were significantly higher in non-survivors than in survivors (4 [2–8] vs. 3 [0–7]; *p <* 0.0001). Survivors had a significantly longer hospitalization time compared to non-survivors (4 [3–7] vs. 2 [1–4] days; *p* = 0.004).

Comparative hematological and biochemical results are shown in Table 2. Compared to survivors, non-survivors had significantly higher neutrophil counts (*p* = 0.001), serum creatinine concentrations (*p <* 0.0001), CAR (*p* = 0.001), NCI (*p <* 0.0001), and NLR (*p* = 0.025) but significantly lower serum albumin concentrations (*p* = 0.017). Lymphocyte counts did not differ significantly between groups (*p* = 0.68).

Analysis of mortality prevalence across biomarker percentiles showed a significant progressive increase in mortality with increasing CAR percentiles (χ^2^ *p* < 0.0001; Somers’ D = 0.282) and NCI percentiles (χ^2^ *p* = 0.001; Somers’ D = 0.312) (Table 3). In contrast, the increase in NLR percentiles was not significantly associated with mortality (χ^2^ *p* = 0.09; Somers’ D = 0.024).

**Figure 2:**
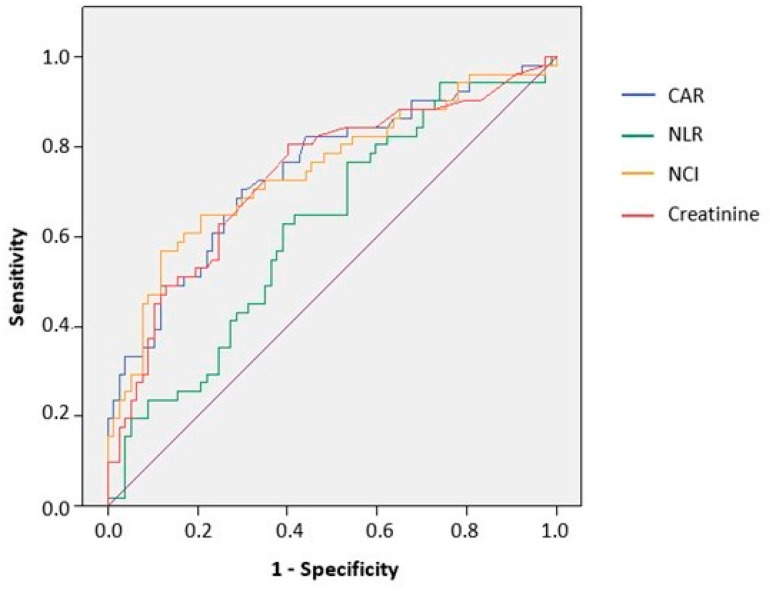
Receiver operating characteristic (ROC) curves comparing the prognostic performance of the creatinine-to-albumin ratio (CAR), neutrophil-to-creatinine index (NCI), serum creatinine, and neutrophil-to-lymphocyte ratio (NLR) for predicting in-hospital mortality in dogs with acute pancreatitis. The purple diagonal line represents the line of no discrimination (AUC = 0.5), corresponding to the performance expected by random chance.

The results of the ROC curve analysis are presented in Table 4 and illustrated in Figure 2. Among the evaluated ratios, CAR and NCI exhibited the highest AUC values (0.747), followed by serum creatinine concentration (0.734). NLR showed the lowest AUC (0.619). Comparison of the ROC curves using the DeLong test revealed no statistically significant differences in the overall discriminatory ability between creatinine and either the CAR (*p* = 0.479) or the NCI (*p* = 0.712), nor between the two composite indices themselves (*p* = 0.982). However, when compared to the traditional NLR, the AUC for the NCI was significantly greater (*p* = 0.021), whereas the difference with the CAR did not reach statistical significance (*p* = 0.060). Creatinine and neutrophil count showed the highest sensitivity, whereas NCI and CAR demonstrated the highest specificity.

Values above the cutoff were associated with increased odds of death for all evaluated markers, with the highest odds ratio observed for NCI, followed by creatinine, CAR, and albumin.

In univariate logistic regression analysis, survival was significantly associated with neutrophil count (OR 0.941; 95% CI 0.899–0.985; *p* = 0.01), serum creatinine levels (OR 0.763; 95% CI 0.685–0.885; *p <* 0.0001), serum albumin concentration (OR 2.165; 95% CI 1.075–4.360; *p* = 0.031), NCI (OR 0.980; 95% CI 0.970–0.991; *p <* 0.0001), CAR (OR 0.385; 95% CI 0.241–0.617; *p <* 0.0001), and NLR (OR 0.966; 95% CI 0.935–0.999; *p* = 0.035).

In multivariable regression models including the NLR and CAR, only the CAR remained independently associated with survival (OR 0.392; 95% CI 0.243–0.632; *p <* 0.001), whereas the NLR was not independently associated with outcome (*p* = 0.197). Similarly, in models including the NLR and NCI, only the NCI retained significance (OR 0.981; 95% CI 0.970–0.991; *p <* 0.001), while the NLR did not (*p* = 0.639).

When the CAR and NCI were included simultaneously in the same model, neither variable remained statistically significant (CAR *p* = 0.199; NCI *p* = 0.200). Likewise, when the NLR, CAR, and NCI were included together, none of the variables remained independently associated with survival (all *p* > 0.05).

In multivariable models assessing the CAR and its components, both creatinine (OR 2.297; 95% CI 1.111–4.750; *p* = 0.025) and CAR (OR 0.034; 95% CI 0.003–0.325; *p* = 0.003) remained independently associated with survival. In the model including the CAR and albumin, only the CAR retained significance (OR 0.384; 95% CI 0.237–0.623; *p <* 0.001), whereas albumin did not (*p* = 0.077). When the CAR, creatinine, and albumin were included simultaneously, only the CAR remained independently associated with survival (OR 0.022; 95% CI 0.001–0.675; *p* = 0.029).

In models evaluating the NCI and its components, only the NCI remained independently associated with survival when adjusted for neutrophil count (OR 0.982; 95% CI 0.971–0.992; *p* = 0.001) or creatinine (OR 0.975; 95% CI 0.954–0.996; *p* = 0.020). When neutrophils, creatinine, and the NCI were included together, none of the variables retained independent significance (all *p* > 0.05).

Correlation analysis showed a moderate positive correlation between the CAR and CAPCSI score (r = 0.447; *p <* 0.0001), a weak positive correlation between the NLR and CAPCSI (r = 0.234; *p* = 0.008), and no significant correlation between the NCI and CAPCSI (*p* = 0.131).

## 4. Discussion

The identification of reliable prognostic markers in AP is essential for the early recognition of high-risk patients and for guiding therapeutic intensity. In the present study, we hypothesized that composite indices integrating biomarkers of inflammation (neutrophils and albumin) and renal function (creatinine) would improve prognostic accuracy compared with isolated analytes or traditional inflammatory indices. Our results partially support this hypothesis. While the NCI and CAR did not provide better overall discriminatory ability than serum creatinine alone for distinguishing survivors from non-survivors, they exhibited greater specificity. Moreover, both composite indices, especially the NCI, outperformed the traditional NLR as predictors of in-hospital mortality in dogs with AP.

The mortality rate observed in this cohort (38%) was higher than the 8.8–33% reported in recent studies [17,21,22] but remained within historically reported ranges of 27–58% [21]. As previously described by our research group [23], this elevated mortality likely reflects the rural referral context of our center, where delayed presentation and advanced disease severity at admission are common. Indeed, non-survivors in the present study exhibited a median of 4 days of clinical signs prior to admission and significantly higher CAPCSI scores at presentation.

The presence of AKI was a prominent feature in our population, affecting 42.5% of dogs, a prevalence consistent with recent reports [24] but exceeding earlier figures of approximately 26% [3]. Exclusion of dogs with known chronic kidney disease, along with the clinical presentation and timing, supports the interpretation that renal dysfunction was secondary to AP rather than pre-existing.

The indices evaluated in this study were designed to integrate complementary pathophysiological domains relevant to AP. Neutrophils play a central role in disease progression through excessive activation and release of proteolytic enzymes, reactive oxygen species, and pro-inflammatory cytokines, thereby amplifying local and systemic tissue damage [4,25]. Albumin, a negative acute-phase protein, decreases in AP due to reprioritization of hepatic protein synthesis and increased vascular permeability induced by inflammation [9,14]. Additional contributors include reduced intake due to abdominal pain and increased catabolic activity driven by inflammatory mediators [13,26]. Renal dysfunction in AP has been attributed to hypovolemia, microcirculatory alterations, endothelial injury, and cytokine-induced oxidative stress [4,27].

To our knowledge, this is the first study to evaluate the prognostic value of the CAR and NCI in canine AP. In human medicine, the CAR has been associated with mortality, postoperative complications, and poor clinical outcomes in patients with AP [12], and with both short- and long-term mortality in critically ill AP patients [11]. The prognostic performance of the CAR in our study (cutoff 0.48; AUC 0.74; sensitivity 70.6%; specificity 70.1%) closely mirrors values reported in human intensive care populations [11], supporting cross-species consistency in its prognostic behavior.

Similarly, the prognostic utility of the NCI has previously been reported only once in humans, where it was identified as an independent predictor of AP severity [15]. Although the cutoff reported in that study (11.27) was lower than ours (26), this discrepancy is likely explained by differences in outcome definition, as our cutoff was established for predicting mortality rather than disease severity. In both human and canine studies, increasing NCI values were associated with more severe clinical presentations and worse outcomes.

The NLR is one of the most extensively studied hematological markers of systemic inflammation [28,29,30,31,32]. Although the NLR was significantly higher in dogs with AP compared with healthy controls and in non-survivors compared with survivors, its prognostic value in canine AP remains inconsistent. While a positive association between NLR percentiles and predicted mortality was reported by Johnson et al. [17], neither the present study nor our previous work [23] identified a significant increase in mortality prevalence across NLR percentiles. Similarly, Guglielmini et al. [7] failed to demonstrate an association between the NLR and mortality. These discrepancies and the lack of difference in the NLR between azotemic and non-azotemic dogs likely reflect the limited ability of the NLR to capture organ dysfunction, a key determinant of outcome in AP.

In contrast, both the CAR and NCI showed consistent associations with outcome across multiple analytical approaches. Higher CAR and NCI percentiles were associated with a higher prevalence of mortality. In ROC curve analysis, both indices yielded higher AUCs than the NLR, with the NCI demonstrating statistically superior discrimination. Furthermore, logistic regression models confirmed their association with survival. Importantly, the CAR and NCI remained independently associated with outcome when adjusted for the NLR, whereas the NLR did not retain significance.

However, when the CAR and NCI were included together in the same multivariable model, neither remained independently associated with survival. This finding may indicate that both indices capture overlapping biological information, primarily related to their shared creatinine component.

We also compared the predictive performance of these ratios with their components.

Creatinine alone has repeatedly been associated with mortality in canine AP [3,9,24], and mortality among azotemic dogs in our study (59.6%) was higher than in non-azotemic dogs and comparable to previous reports [24,33]. In the ROC analysis, creatinine showed a similar predictive ability to the CAR and NCI to differentiate mortality and survival, and better than the NLR. However, while creatinine showed good sensitivity for mortality prediction, its specificity was limited, potentially leading to risk overestimation. CAR and NCI, by contrast, demonstrated higher specificity and a more balanced sensitivity-specificity profile, acting as more reliable markers to confirm poor prognosis and reducing the false-positive rate associated with creatinine alone. In multivariate analysis including the NCI and creatinine, the index was the only independent predictor. Because the CAR and NCI are derived from routinely available laboratory variables, they may represent practical and inexpensive tools for early risk stratification in dogs with AP.

Albumin performed worse than the CAR in terms of forecasting mortality in AP dogs, showing the second-lowest AUC of all the analytes. Multivariable analyses confirmed that the CAR remained independently associated with survival after adjustment for albumin and creatinine, whereas albumin alone did not consistently retain significance. This finding is consistent with the possibility that the CAR captures prognostic information beyond isolated hypoalbuminemia, which may be influenced by nutritional status or chronic inflammatory conditions.

Although the neutrophil count is useful in distinguishing mortality from survival, the AUC and odds ratio of death in dogs over the NCI cutoff were superior to those of the neutrophil count. The NCI remained independently associated with survival after adjustment for neutrophil count or creatinine, supporting its potential value as a composite marker integrating inflammatory and renal variables.

Finally, regarding the CAPCSI, the CAR showed a moderate positive correlation with clinical severity, whereas the NCI did not. This dissociation may reflect differences between the clinical variables captured by the CAPCSI and the laboratory variables incorporated into the NCI.

Several limitations should be acknowledged. To the best of our knowledge, this is the first manuscript to evaluate the prognostic utility of the CAR and NCI in veterinary medicine. Consequently, while the observed trends align with reports in human literature, the direct extrapolation of quantitative findings requires caution. Inherent interspecies differences in basal hematological and biochemical profiles [34,35], combined with the variability in the clinical stage of animals at the time of admission, inevitably influence the operational ranges of these biomarkers compared to human cohorts. Because of the retrospective design, a standardized physiological fasting status prior to blood collection could not be guaranteed due to the patients’ acute presentation (e.g., vomiting, anorexia), and treatments administered after admission were not strictly standardized.

This was a retrospective, single-center study with a relatively limited sample size, particularly with regard to the number of non-survivors. Therefore, although the results support the potential prognostic utility of the CAR and NCI in dogs with acute pancreatitis, the proposed cutoff values should be interpreted with caution until they are confirmed in larger, prospective, multicenter studies and independent validation cohorts. Further studies are warranted to assess the robustness, reproducibility, and generalizability of these findings.

Although cPL is a sensitive diagnostic tool, the absence of histopathological confirmation means that misclassification of other acute abdominal disorders cannot be fully excluded. Additionally, while dogs with known chronic kidney disease were excluded, the presence of subclinical renal disease cannot be entirely ruled out. Another inherent limitation is the combined mortality endpoint of natural death and euthanasia, which can introduce clinical decision bias. Nevertheless, this bias was mitigated in our cohort as euthanasia was strictly chosen based on terminal clinical criteria rather than financial constraints. Finally, although a healthy control group was included to establish baseline ratios, and the values of their individual analytes and the NLR aligned with established physiological reference intervals and previous veterinary literature [28,36,37], these dogs were significantly younger than the AP cohort. Because age can independently influence serum albumin concentrations, renal biomarkers, and leukocyte profiles, future studies should aim to use age-matched controls to minimize demographic bias.

## 5. Conclusions

Creatinine-derived composite indices were associated with outcome and showed potential prognostic utility in dogs with acute pancreatitis. The creatinine-to-albumin ratio (CAR) and the neutrophil-to-creatinine index (NCI) were significantly associated with in-hospital mortality. Notably, the NCI demonstrated statistically superior prognostic performance compared to the traditional neutrophil-to-lymphocyte ratio (NLR).

Because the CAR and NCI are derived from routinely available laboratory parameters associated with systemic inflammation and renal dysfunction, they may provide clinically useful prognostic information for early risk stratification and support clinical decision-making in dogs with acute pancreatitis.

## Figures and Tables

**Table 1:** Biomarkers of control group (healthy dogs) and dogs with acute pancreatitis.

	Control Group (n = 40)	AP Dogs (n = 134)	*p*-Value
Neutrophils (×10^3^ μL)	5.99 (4.12–10.54)	13.52 (1.05–49.84)	<0.0001
Lymphocytes (×10^3^ μL)	2.76 (1–4)	1.10 (0–7)	<0.0001
Albumin (mg/dL)	3.80 (3–4)	2.45 (1–5)	<0.0001
Creatinine (mg/dL)	1 (0.7–1.1)	1.2 (0.3–12)	<0.0001
CAR	0.28 (0.16–0.34)	0.44 (0.11–10)	<0.0001
NCI	5.6 (4.75–7.15)	19.16 (0.06–305.28)	<0.0001
NLR	2.86 (1.16–6.14)	8.85 (0.15–61.7)	<0.0001

AP: acute pancreatitis; CAR: creatinine-to-albumin ratio; NCI: neutrophil-to-creatinine index; NLR: neutrophil-to-lymphocyte ratio. Data are shown as median and range (min–max).

**Table 2:** Hematological-derived marker results for survivors and non-survivors with acute pancreatitis.

	Survivors (n = 83)	Non-Survivors (n = 51)	*p*-Value
Neutrophils (×10^3^ μL)	11.12 (0.27–49.57)	16.92 (0.05–44.53)	0.001
Lymphocytes (×10^3^ μL)	1.29 (0–7)	1.10 (0–6)	0.68
Albumin (mg/dL)	2.97 (1–5)	2.52 (2–4)	0.017
Creatinine (mg/dL)	1.1 (0.3–10.5)	2.9 (0.5–12)	<0.0001
CAR	0.37 (0.11–2.76)	1.05 (0.14–10)	0.001
NCI	12.48 (0.22–156.66)	42.73 (0.06–305.28)	<0.0001
NLR	8.99 (0.15–47.29)	11.23 (0.4–61.29)	0.025

CAR: creatinine-to-albumin ratio; NCI: neutrophil-to-creatinine index; NLR: neutrophil-to-lymphocyte ratio. Data are shown as median and range (min–max).

**Table 3:** Prevalence of death by marker percentiles in dogs with acute pancreatitis.

		Percentile 25	Percentile 50	Percentile 75	Percentile 90	*p*-Value
CAR	Value	0.32	0.44	1.23	2.72	χ^2^ < 0.0001 *D*’s 0.282
	% death (95%Cl) n	21.9 (9.3–40) 7	21.9 (9.3–40) 7	43.8 (26.4–62.3) 14	71.9 (53.3–86.3) 23	
NCI	Value	9.1	19.2	46.8	129.85	χ^2^ 0.001 *D*’s 0.312
	% death (95%Cl) n	18.7 (7.2–36.4) 6	25 (11.5–43.4) 8	43.8 (26.4–62.3) 14	71.9 (53.3–86.3) 23	
NLR	Value % death (95%Cl) n	5.59 25 (11.5–43.4) 8	9.84 34.4 (18.6–53.2) 11	17.72 53.1 (34.7–70.9) 17	29.59 46.9 (29.1–65.3) 15	χ^2^ 0.09 *D*’s 0.024

*p*-values correspond to chi-squared test (χ^2^) and Somers’ D (*D*) test. CAR: creatinine-to-albumin ratio; NCI: neutrophil-to-creatinine index; NLR: neutrophil-to-lymphocyte ratio.

**Table 4:** Receiver operating characteristic (ROC) curve analysis, showing the overall accuracies of the biomarkers used to distinguish survivors from non-survivors in all dogs with acute pancreatitis.

	AUC (95% CI)	*p*	Cutoff	Sensitivity (%)	Specificity (%)	Odds Ratio (95% CI)	*p*
Neutrophils (×10^3^ μL)	0.652 (0.565–0.80)	0.0002	12.67	70.5 (54.1–79.5)	56.2 (45.5–66.7)	2.60 (1.15–6.20)	<0.0001
Creatinine (mg/dL)	0.734 (0.642–0.825)	<0.0001	1.19	80.39 (67.3–89.1)	40.25 (48.6–70)	6.08 (2.66–13.92)	<0.0001
Albumin (g/dL)	0.642 (0.525–0.727)	0.015	2.71	69.5 (52.2–74.1)	50.5 (40.8–66.6)	3.30 (1.12–7.56)	0.006
CAR	0.747 (0.658–0.853)	<0.0001	0.48	70.6 (56.9–81.3)	70.1 (59.1–79.2)	5.63 (2.59–12.23)	<0.0001
NCI	0.747 (0.649–0.831)	<0.0001	26	65.4 (51.8– 76.8)	78.9 (68.5–86.6)	7.08 (3.20–15.67)	<0.0001
NLR	0.619 (0.416–0.725)	0.020	10.35	60.5 (49.7–73.2)	60.0 (48.8–70.6)	2.64 (1.27–6.47)	0.002

AUC: area under the receiver operating characteristic curve, CAR: creatinine-to-albumin ratio, NCI: neutrophil-to-creatinine index, NLR: neutrophil-to-lymphocyte ratio.

## Data Availability

The data used in this study were obtained from veterinary clinical management software and belong to the participating veterinary hospital. Due to confidentiality and privacy policies, the datasets are not publicly available. Anonymized data may be provided by the corresponding author on reasonable request and with the approval of the data owner.

## Supplementary Materials

The following supporting information can be downloaded at: https://www.mdpi.com/article/10.3390/ani16152283/s1, Table S1: Biomarkers of azotemic dogs and non-azotemic dogs with acute pancreatitis.

## Abbreviations

The following abbreviations are used in this manuscript:

AP	Acute pancreatitis
AKI	Acute kidney injury
CAPCSI	Canine acute pancreatitis clinical severity index
CAR	Creatinine-to-albumin ratio
NCI	Neutrophil–creatinine index
NLR	Neutrophil-to-lymphocyte ratio
