# Supplementary material for: Creatinine-Derived Composite Indices for Improved Mortality Prediction in Dogs with Acute Pancreatitis
Source: Animals (Basel). 2026 Jul 23;16(15):2283. doi: 10.3390/ani16152283 (PMC13463579; doi:10.3390/ani16152283)
Supplement: Supplementary file 1 [file animals-16-02283-s001.zip › animals-4397798-supplementary.pdf]

animals

MDPI

Table S1. Biomarkers of azotemic dogs and non-azotemic dogs with acute pancreatitis.

	**Azotemic dogs (n = 57)**	**Non-azotemic dogs (n = 77)**	***p*-Value**
Age (years)	11 (3–18)	10 (1–18)	< 0.0001
Sex (Male/female)	29/23	43/33	0.928
Mortality rate	34/57 (59.6%)	20/77 (25.9%)	< 0.0001
Neutrophils ($\times 10^3$ µL)	13.69 (0.63–44.53)	12.57 (0.05–49.79)	0.379
Lymphocytes ($\times 10^3$ µL)	1.29 (0–7)	1.31 (0–6)	0.959
Albumin (g/dL)	2.8 (2–5)	2.73 (1–4)	0.93
Creatinine (mg/dL)	4.3 (1.7–12)	1 (0.3–1.1)	< 0.0001
CAR	1.6 (0.34–10)	0.343 (0.11–0.7)	< 0.0001
NCI	62.33 (2.39–305.28)	10.05 (0.06–49.84)	< 0.0001
NLR	9.57 (0.4–61.7)	10.12 (0.15–47.29)	0.587

CAR: creatinine-to-albumin ratio; NCI: neutrophil-to-creatinine index; NLR: neutrophil-to-lymphocytes ratio. Data are shown as median and range (min-max).
